# Reevaluating Pediatric Osteomyelitis with Osteoarticular Tuberculosis: Addressing Diagnostic Delays and Improving Treatment Outcomes

**DOI:** 10.3390/children11111279

**Published:** 2024-10-23

**Authors:** Alexandru Herdea, Harun Marie, Ioana-Alexandra Negrila, Aliss Delia Abdel Hamid Ahmed, Alexandru Ulici

**Affiliations:** 1 11th Department of Pediatric Orthopedics, “Carol Davila” University of Medicine and Pharmacy, Bd. Eroii Sanitari Nr. 8, 050474 Bucharest, Romania; alexandru.herdea@umfcd.ro (A.H.); alexandru.ulici@umfcd.ro (A.U.); 2 Pediatric Orthopedics Department, “Grigore Alexandrescu” Children’s Emergency Hospital, 011743 Bucharest, Romania

**Keywords:** pediatric osteoarticular tuberculosis, tuberculous osteomyelitis, multimodal diagnosis, BCG vaccination, diagnostic challenges, surgical intervention, anti-TB therapy, long-term complications, multidisciplinary management, early detection and treatment

## Abstract

Background: Pediatric osteoarticular tuberculosis (TB) remains a significant global health challenge, particularly in resource-limited settings, where delayed diagnosis and treatment frequently lead to severe long-term complications. Despite advancements in TB control, skeletal TB in children is often misdiagnosed due to its non-specific clinical presentation, contributing to poor outcomes such as joint deformities, growth disturbances, and chronic pain. The complexity of diagnosing osteoarticular TB is further exacerbated by the limited sensitivity of conventional diagnostic tools and the overlap with other musculoskeletal conditions. This study seeks to evaluate the impact of early detection and multidisciplinary management on treatment outcomes in pediatric patients with osteoarticular TB. Methods: A retrospective review was conducted at the Pediatric Orthopedics Department of the “Grigore Alexandrescu” Children’s Hospital in Romania from 2009 to 2023. Case data included clinical, imaging, and microbiological findings, and treatment outcomes in children aged 0–18 years diagnosed with tuberculous osteomyelitis. Results: The study identified varied clinical presentations, with delayed diagnosis often linked to misinterpretation of symptoms as non-TB infections. Multimodal diagnostic approaches combining imaging, microbiological testing, and histopathology improved diagnostic accuracy. Early surgical intervention alongside anti-TB therapy proved effective in reducing long-term complications. Conclusions: Timely, accurate diagnosis and multidisciplinary treatment are critical to improving outcomes in pediatric osteoarticular TB. Vaccination status and comprehensive diagnostic tools significantly influence disease progression and treatment success. The study underscores the need for enhanced screening and diagnostic methods to prevent delays in treatment.

## 1. Introduction

Osteoarticular tuberculosis (TB), caused by *Mycobacterium tuberculosis* (MT), remains a chronic and debilitating infection that poses a significant global health challenge, particularly in developing countries [1]. While pulmonary TB is more widely recognized, the disease can also affect the skeletal system, with children being particularly vulnerable. Pediatric cases are often more severe and difficult to diagnose, as symptoms can differ significantly from those in adults [2,3], and bony lesions may not be easily detected on standard X-rays. Additionally, the focus on childhood TB in clinical practice and public health policy remains insufficient, contributing to delays in diagnosis and treatment [3,4].

Children represent a critical demographic in TB control efforts, as they contribute to the future reservoir of active TB cases and suffer significant morbidity and mortality rates. Effective strategies to mitigate this include early risk-based screening, chemoprophylaxis for those at high risk, and prompt administration of the Bacillus Calmette–Guérin (BCG) vaccine soon after birth [5]. Adolescents, who often face unique psychosocial challenges and may struggle with treatment adherence, also require specific attention in TB management guidelines [6].

Despite concerted efforts to control TB, delayed diagnosis of tuberculous osteomyelitis can result in severe complications, including permanent deformities and neurological deficits. Identifying at-risk populations and understanding the varied clinical and radiological presentations of musculoskeletal TB are essential for timely diagnosis and treatment [7]. The disease’s heterogeneity can lead to confusion with other conditions such as fungal infections and bone tumors, further emphasizing the need for accurate diagnostic approaches [3,8,9].

The historical context of osteoarticular TB, including Pott’s disease and the discovery of Koch’s bacillus, provides a foundation for understanding the disease’s progression and management. The identification of MT by Robert Koch in 1882 revolutionized our understanding of TB, paving the way for the development of antibiotics and the BCG vaccine, which remain central to TB control efforts today [10,11,12].

Recent studies emphasize that early diagnosis of extrapulmonary TB, especially in children, relies heavily on advanced molecular diagnostics like Xpert MTB/RIF, which can significantly improve detection rates in resource-constrained settings. BCG vaccination continues to play a critical role in reducing the burden of severe TB in children, though its efficacy in preventing osteoarticular TB remains a topic of discussion [1,11,13,14].

Disseminated TB, including osteoarticular involvement, continues to be a significant public health concern, especially in high-burden countries [15]. According to the WHO Global Tuberculosis Report 2023, 10.6 million people worldwide fell ill with tuberculosis (TB) in 2022, marking a slight increase from the previous year. TB remains one of the top infectious disease killers globally, accounting for approximately 1.3 million deaths in 2022. Extrapulmonary TB, which includes skeletal TB, is known to affect between 10 and 20% of TB patients globally, and pediatric cases represent around 11–12% of the total global tuberculosis burden. Countries with high TB burdens, such as India, Indonesia, and parts of Africa, often see higher rates of pediatric tuberculosis, with skeletal TB being a significant part of the extrapulmonary burden [16].

This retrospective study provides a comprehensive narrative of the literature and clinical case evaluations regarding pediatric osteoarticular TB and aims to evaluate the impact of vaccination status on the occurrence of tuberculous osteomyelitis; assess current diagnostic, preventive, and treatment methods; identify affected osteoarticular segments; and examine complications resulting from tuberculous osteomyelitis in children. This study seeks to explore how a multidisciplinary approach, combined with advanced diagnostic techniques, can improve early diagnosis, treatment outcomes, and long-term management of pediatric skeletal tuberculosis. Specifically, the research aims to evaluate the impact of multidisciplinary collaboration on enhancing diagnostic accuracy and reducing delays in pediatric cases. Furthermore, the study assesses the role of advanced diagnostic tools, such as arthroscopy and immunological testing, in differentiating skeletal tuberculosis from osteomyelitis. It also investigates the long-term outcomes following early diagnosis and intervention, while considering the socioeconomic factors influencing access to care and contributing to disparities in treatment outcomes across different patient populations.

## 2. Materials and Methods

### 2.1. Study Design

The study was conducted at the Pediatric Orthopedics Outpatient Clinic of the “Grigore Alexandrescu” Children’s Emergency Clinical Hospital in Bucharest, Romania. This facility serves children from the urban vicinity and surrounding areas. Ethical approval was obtained from the hospital’s ethics committee (no. 10/11 April 2024). Informed consent was secured from the parents or legal guardians of all participants. The study spanned the period from 2009 to December 2023, and included literature search strategies employing relevant English keywords such as “TB”, “skeletal”, “bone”, “osteomyelitis”, “child”, “pediatric”, and “orthopedics”.

#### 2.1.1. Case Studies

This retrospective study included children aged from newborn to 18 years diagnosed with tuberculous osteomyelitis. Inclusion criteria were confirmed diagnoses based on clinical, laboratory, imaging (X-rays, MRI, or CT scans), and microbiological data (positive culture or PCR for MT). Exclusion criteria included incomplete data or patient history, follow-up duration of less than 2 years, presence of local comorbidities, and studies including adults.

#### 2.1.2. Theoretical Framework

Only articles meeting specific criteria were considered: they had to involve studies conducted on human subjects, be written in English, and focus on children (aged newborn to 18 years). Specifically, articles exclusively addressing bone TB in pediatric orthopedics were chosen. Participants were required to have a minimum follow-up period of 2 years. Exclusion criteria regarding the selection of scientific articles concerning pediatric tuberculous osteomyelitis included the following: articles that focused on the adult population (over the age of 18 years), articles not written in English, studies concerning animal studies or in vitro experiments. A minimum follow-up period of two years was established to ensure the thorough assessment of long-term treatment outcomes in pediatric osteoarticular tuberculosis (TB). Osteoarticular TB is a chronic disease that often requires extended treatment and recovery periods. A two-year follow-up allows for the observation of critical outcomes such as bone healing, growth disturbances, and potential relapses, which may not manifest in shorter time frames. This duration also aligns with clinical guidelines, which recommend extended monitoring to detect late complications and ensure robust data on treatment efficacy. Excluding cases with shorter follow-up periods helps minimize bias and enhances the reliability of the study’s conclusions. Furthermore, case reports were excluded, and only articles analyzing a minimum of 15 patients were included. Following the criteria described above, we obtained 16 articles that had all the mentioned characteristics (Table 1).

### 2.2. Study Procedure

Following a comprehensive collection of medical history and a thorough clinical examination relating to osteoarticular TB, imaging studies such as X-rays, MRI, and/or CT scans were conducted to confirm the diagnosis and evaluate the extent of the disease. Laboratory investigations, including complete blood count, erythrocyte sedimentation rate (ESR), C-reactive protein (CRP), and a tuberculin skin test or interferon-gamma release assay (IGRA), were also performed. The management of tuberculous osteomyelitis involved a combination of pharmacotherapy and surgical intervention. Anti-TB medications were administered in accordance with the national TB treatment guidelines, which consisted of a multidrug regimen comprising isoniazid, rifampicin, pyrazinamide, and ethambutol, with or without streptomycin. Surgical intervention, such as debridement, sequestrectomy, or bone grafting, was conducted when deemed necessary based on the disease’s extent and the presence of complications. Follow-up assessments were carried out at regular intervals, involving clinical examination, imaging studies, and laboratory investigations, to monitor treatment response, assess disease progression, and detect any complications. The minimum follow-up duration for each patient was 2 years.

### 2.3. Data Collection and Analysis

Data were collected and stored in the institutional informatics system. Filters were applied to select articles that met specific criteria: they had to involve studies conducted on human subjects, be written in English, and focus on children (aged newborn to 18 years). Only articles exclusively addressing bone TB in pediatric orthopedics were chosen. Studies including the adult population were excluded. Additionally, case reports were not considered, and only articles analyzing a minimum of 15 patients were included. Further details regarding data analysis were not provided. Descriptive statistics were used to summarize patient demographics, clinical presentations, and follow-up outcomes. Comparative analyses, such as chi-square tests and *t*-tests, were applied to evaluate differences between subgroups, such as vaccinated versus unvaccinated patients, and to assess the significance of treatment outcomes. These methods were selected based on their appropriateness for categorical and continuous data, ensuring a clear understanding of the relationships between variables. By utilizing these statistical techniques, we aimed to provide robust, transparent, and reproducible results that reflect the real-world complexity of TB in pediatric populations.

### 2.4. Diagnostic Methods

Diagnosing pediatric osteo-articular TB is complex due to non-specific symptoms and varied clinical presentations, from unnoticed infection to severe health impacts. The diagnostic process relies on correlating historical, clinical, imaging, and laboratory data.

Symptoms often include pain, swelling, and limited movement, and severe cases might involve neural impairments like paraparesis. Systemic symptoms such as weight loss, fever, and fatigue may also occur but are not consistent. Diagnostic clarity may improve with a history of exposure to TB or prior infections suggesting reactivation. Imaging and laboratory tests, highlighting inflammatory markers, are essential in reinforcing suspected diagnoses. Specific tests like IGRA or skin tests, and confirmatory methods such as biopsies revealing granulomatous tissue, PCR tests, cultures, and special staining, are crucial.

While this study highlights the use of various diagnostic methods, it is important to acknowledge their limitations, particularly in pediatric TB cases. Imaging techniques such as X-rays and MRI, though invaluable, can demonstrate variable sensitivity and specificity. X-rays may fail to detect early-stage bone lesions, while MRI, despite its high resolution, can sometimes produce findings that overlap with other conditions like osteomyelitis or malignancies, leading to potential misdiagnoses. Additionally, in pediatric patients, the growth and developmental stages of bones may complicate the interpretation of imaging results. These limitations emphasize the need for a multimodal diagnostic approach, incorporating clinical, microbiological, and advanced molecular tools to improve diagnostic accuracy. Continued refinement of diagnostic protocols, including the integration of more sensitive molecular assays, is crucial for early and precise identification of TB in children.

### 2.5. Treatment

Effective treatment of bone TB involves a combination of anti-TB medications and, in some cases, surgical interventions. The medications typically include a multidrug regimen (isoniazid, rifampicin, pyrazinamide, ethambutol, with or without streptomycin). Medical strategies must always additionally focus on the patient’s nutritional status. Surgical procedures are employed for diagnostic purposes, to remove necrotic tissue, relieve spinal cord compression, or stabilize the spine [32]. Recent studies suggest that early surgical intervention may improve outcomes by preventing complications such as deformities and neurological deficits [33].

### 2.6. Complications

Regular monitoring of pediatric patients with bone TB is crucial for early detection and management of potential relapses or complications. Post-surgery check-ups typically occur at 6 weeks, 3 months, 9 months, and 1 year. Ongoing annual evaluations are recommended until the child’s skeletal system is fully developed. Early and accurate diagnosis, along with proper treatment, are essential for reducing complications and improving health outcomes. While many patients recover without lasting effects, some may experience less favorable outcomes, including the following:

- Recurring tuberculous abscesses;
- Impaired bone growth leading to skeletal asymmetry;
- Residual joint issues such as arthritis, stiffness, ankylosis, or deformities;
- Hip dislocation or pathological subluxation;
- Spinal deformities or progression of such deformities in cases of Pott’s disease;
- Severe neurological complications, which are among the most serious.

Severe forms of TB, such as miliary TB, continue to be life-threatening, even in otherwise healthy pediatric populations [34,35]. The risk of complications is heightened by drug-resistant TB strains, malnutrition, and poor adherence to treatment protocols. Additionally, the potential side effects of anti-TB medications affecting the kidneys, liver, and eyes should also be considered.

## 3. Clinical Case Presentation

### 3.1. Case 1

A 12-year-old male, referred to as R.M. and BCG-vaccinated, was admitted to the emergency department on 26 October 2020, due to persistent left hip pain and functional limitations that had developed over the preceding year after a bicycle accident. Clinical evaluation identified a limp and exacerbated pain with movement of the left lower extremity. Initial symptomatic relief was noted with rest and oral anti-inflammatory medications.

**Figure 1:**
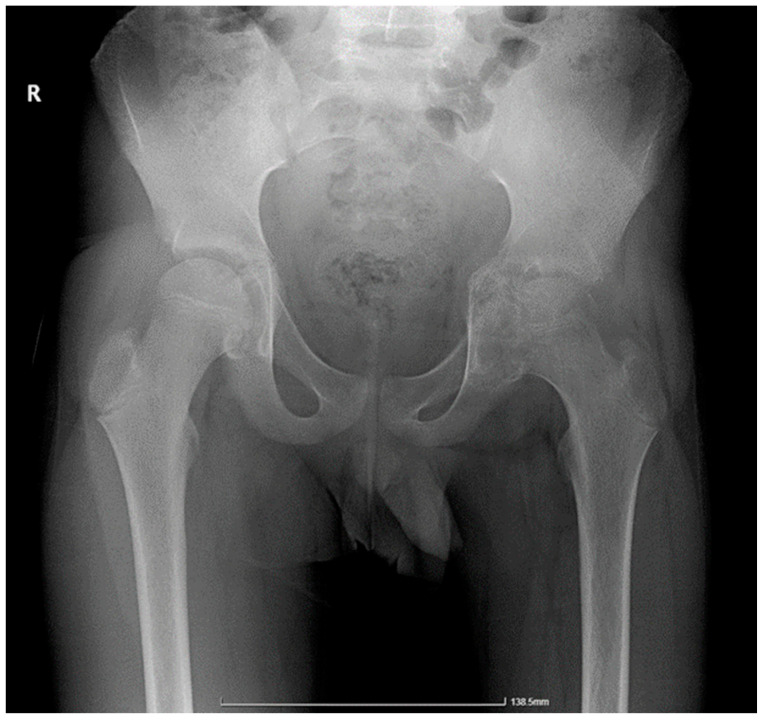
Pelvic radiography: areas of osteolysis at the level of the left hip joint with a decreased joint space, along with thinning and erosion of the joint contours. R indicates the right side of the parient.

**Figure 2:**
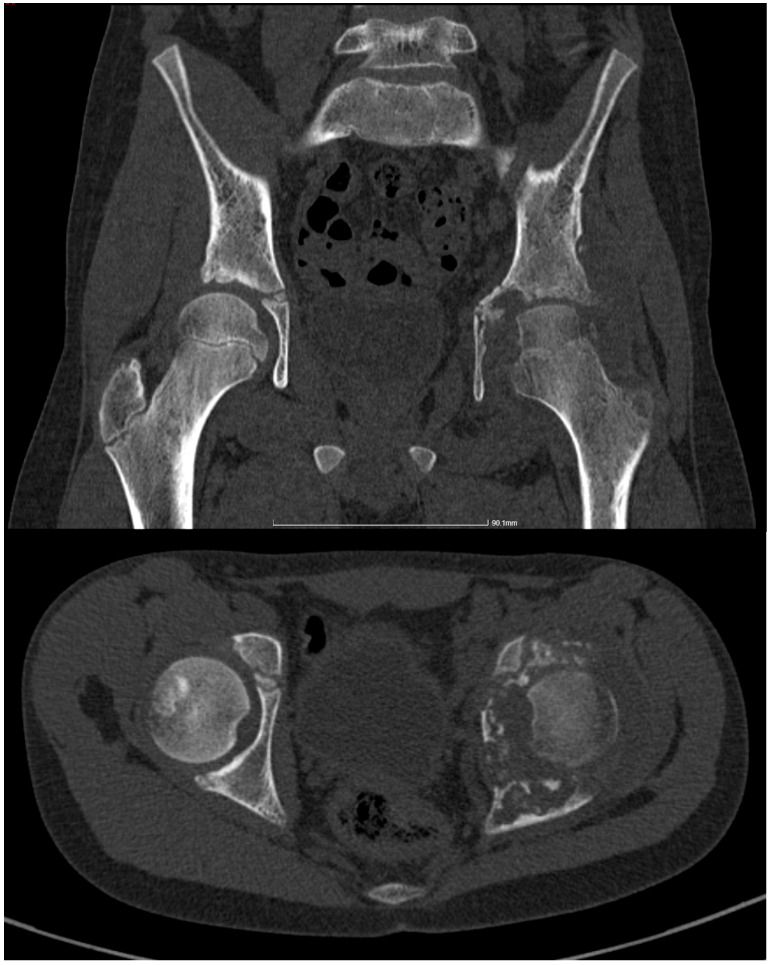
CT scan of the pelvis, cross-sectional and frontal views: extensive area of osteolysis and fragmentation, slightly expansive at the left acetabular and periacetabular level; multiple areas of osteolysis at the left femoral head with no periosteal reaction in the vicinity; intra-articular overflow in the left hip joint of approximately 15 mm, with parafluid densities and calcifications in the joint capsule, in the upper portion.

Diagnostic imaging revealed neoplastic lesions in the left acetabulum and femoral head, prompting urgent referral to Pediatric Orthopedics (Figure 1). Laboratory analyses indicated anemia and elevated inflammatory markers, and screenings for TB including a tuberculin skin test and QuantiFERON test were conducted. Further diagnostic procedures included a chest X-ray, a CT scan of the pelvis (Figure 2), and targeted bone and joint fluid biopsies under general anesthesia, which suggested acute osteomyelitis. The patient was started on intravenous ceftriaxone and rifampicin.

**Figure 3:**
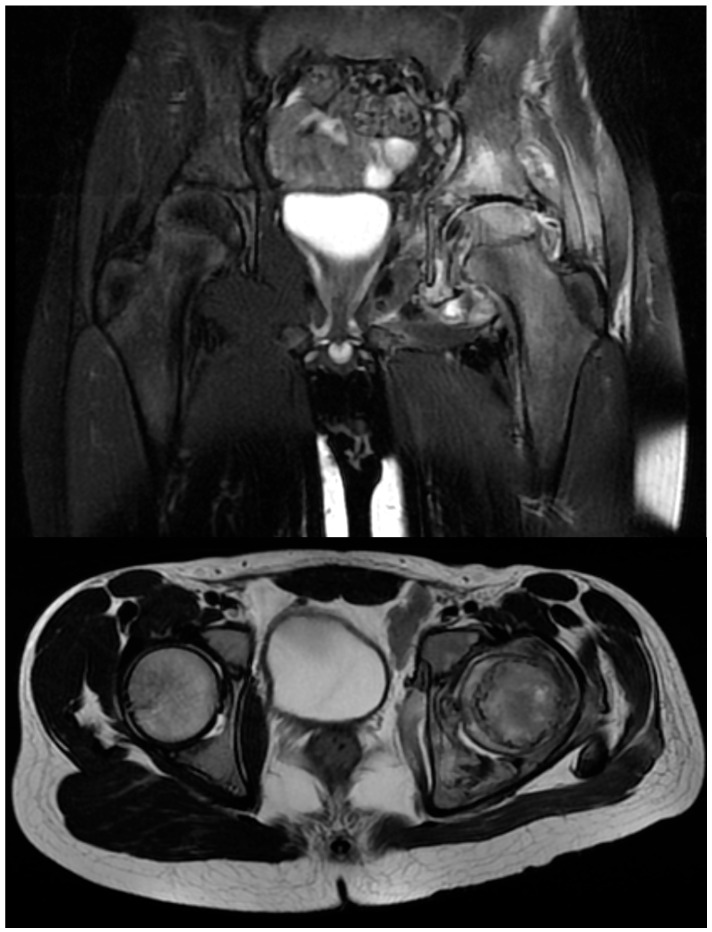
The pelvis MRI shows multiple osteoarticular changes, especially in the left ilium, ischium, and left femoral head, extending towards the neck. These areas display intense inhomogeneous T2 hypersignal and gadolinophilia, suggesting osteomyelitis. There is also an increase in the coxal bone diameter, loss of cortical continuity, uneven thinning of coxo-femoral cartilage, synovial hyperemia and hypertrophy, and increased septic synovial effusion. The infection extends into the gluteal regions and subcutaneous tissue, with significant periarticular soft tissue edema and notable left peri-iliac and peri-femoral lymphadenopathy.

Subsequent consultations led to an incisional biopsy of the left hip joint capsule, showing chronic inflammation and necrotic patches. However, microbial cultures were negative. The patient received intravenous antibiotic treatment starting from 29 October 2020, with Cefort 1 g every 12 h until 10 November 2020, and rifampicin 150 mg until 5 November 2020. This antibiotic regimen was initiated following the clinical suspicion of osteoarticular infection, while awaiting confirmation of the diagnosis. The conclusive diagnosis was osteoarticular TB, confirmed by a pneumophthisiology specialist. On 4 November 2020, the patient underwent a new imaging investigation, specifically an MRI (Figure 3), which provided further insights into the extent of the osteoarticular changes.

Treatment was adjusted and monitored by the pneumoftiziologist to include a comprehensive four-drug antituberculous regimen (isoniazid, rifampicin, pyrazinamide, and ethambutol).The recommended treatment regimen for pediatric osteoarticular tuberculosis followed the national guidelines, which include the use of a combination of first-line anti-TB medications. The patient was treated with isoniazid at a dose of 5 mg/kg body weight per day (up to a maximum of 300 mg), rifampicin at 10 mg/kg body weight per day (up to a maximum of 600 mg), ethambutol at 15 mg/kg body weight per day (up to a maximum of 1600 mg), and pyrazinamide at 25 mg/kg body weight per day (up to a maximum of 2000 mg). These medications were administered daily, as recommended by the guidelines, and the doses were carefully adjusted based on the patient’s weight to ensure effective treatment while minimizing the risk of adverse effects. Despite being discharged against medical advice in a stable condition, the patient engaged in regular follow-ups, showing positive outcomes. Non-weight-bearing physical therapy was initiated after suture removal.

The patient was readmitted on 17 February 2021, with new symptoms in the hip and knee, raising concerns of possible TB dissemination. An MRI indicated reactive changes but no extensive joint or bone damage. After temporary immobilization, the initial suspicion of knee TB was reconsidered as symptoms improved. 

On 5 April 2021, a follow-up revealed a new fistula at the hip, linked to tuberculous arthritis, which had disabled the joint functionally. Both the patient and their guardian were informed about the urgency and necessity of the surgical intervention, along with the associated risks and benefits, as well as the risks of delaying the procedure. However, the guardian refused hospitalization against medical advice, and the local TB dispensary responsible for the patient’s care was informed by phone. The patient returned two days later. Surgical management included debridement and fistula excision on 7 April 2021, although no infectious agents were identified in the samples. Histopathology showed chronic granulomatous inflammation consistent with TB. 

The patient continued on the multidrug therapy, under close surveillance to monitor treatment efficacy and adherence. This case underscores the critical role of a multidisciplinary team in managing complex cases of osteoarticular TB.

### 3.2. Case 2

**Figure 4:**
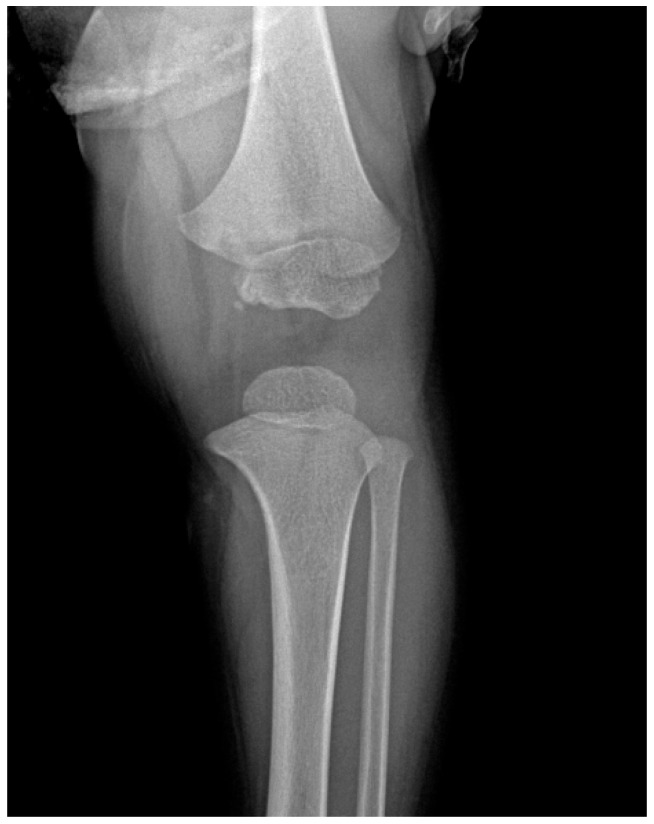
X-ray of the left knee reveals mixed osteocondensation and osteolytic areas in the distal third of the left femur. Additionally, there is evidence of soft tissue inflammation medial to the distal end of the left femur.

**Figure 5:**
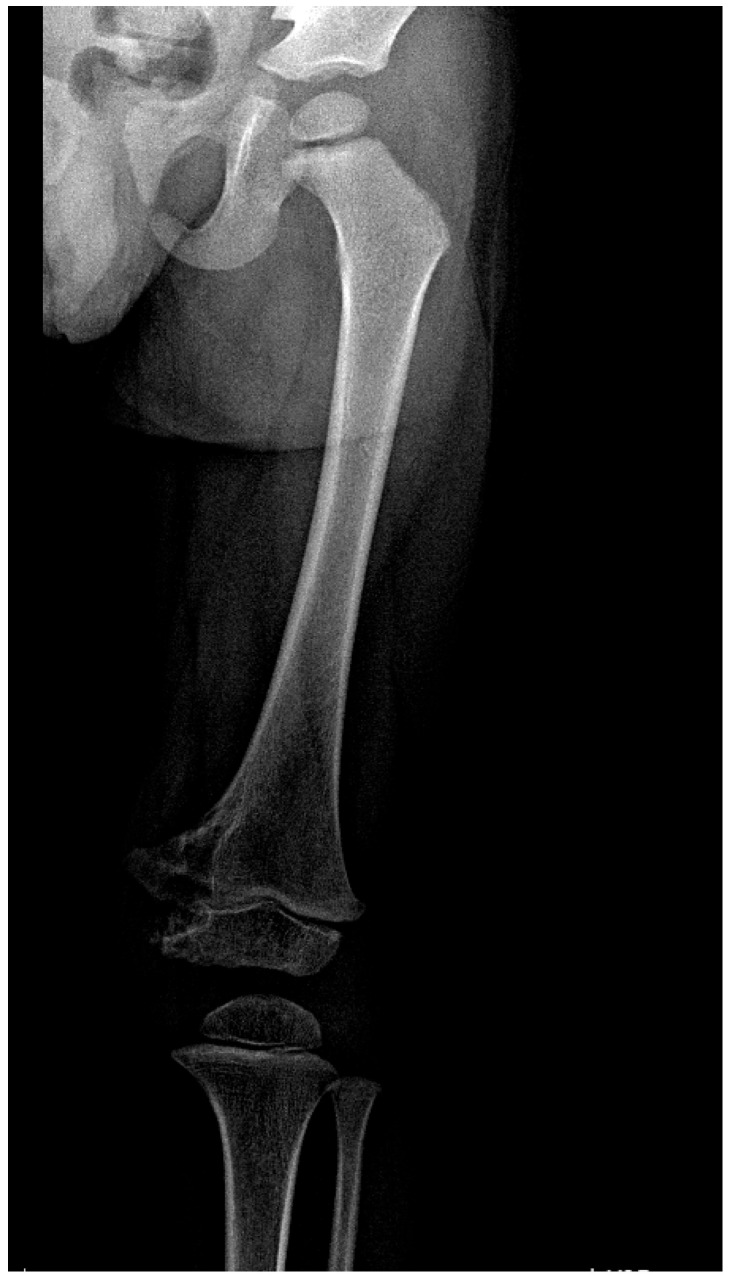
X-ray of the left knee reveals mixed osteocondensing and osteolytic regions in the distal third of the left femur, with the coxo-femoral joint remaining intact.

A male patient, R.M., aged 1 year and 11 months and BCG-vaccinated, presented to the emergency department with an injury to his left knee; after clinical and radiological examination, the patient was diagnosed as having a contusion accompanied by a local hematoma (Figure 4). Initial management involved immobilization with a femoro-pedal cast. Despite removal of the cast after a few days due to persistent local swelling and subsequent reapplication of a new cast, the condition initially improved. However, seven months later, the child returned with a superinfected hematoma in the distal thigh necessitating aspiration and recommendations for maintaining cleanliness and minimizing further trauma and a new radiological examination was carried out (Figure 5).

The following day, acute exacerbation of chronic osteomyelitis in the distal femur led to the patient’s hospitalization. Surgical procedures including incision, irrigation, and drainage of the associated abscess were undertaken. Microbiological tests failed to identify any pathogenic organisms or acid-fast bacilli, while histopathological examination established a clear diagnosis of florid chronic osteomyelitis. Clinical interpretation was advised to consider potential infection with MT.

Subsequent follow-ups involved alternating cast removal and reapplication, showing general improvement. A positive QuantiFERON test later confirmed TB infection, prompting referral to a TB-specialized facility for appropriate management. The patient was treated with a combination of isoniazid, rifampicin, pyrazinamide, and ethambutol, with dosage calculated according to the national guideline as presented in case 1. Follow-up assessments indicated stable radiological findings and positive clinical responses.

One year post-surgery, the patient was actively receiving anti-TB therapy and exhibited promising local recovery. Continuous periodic evaluations were advised to monitor growth and overall progress. Later follow-ups continued to show favorable outcomes without any signs of joint deformity or disease reactivation, underscoring the need for ongoing surveillance to ensure sustained improvement.

### 3.3. Case 3

**Figure 6:**
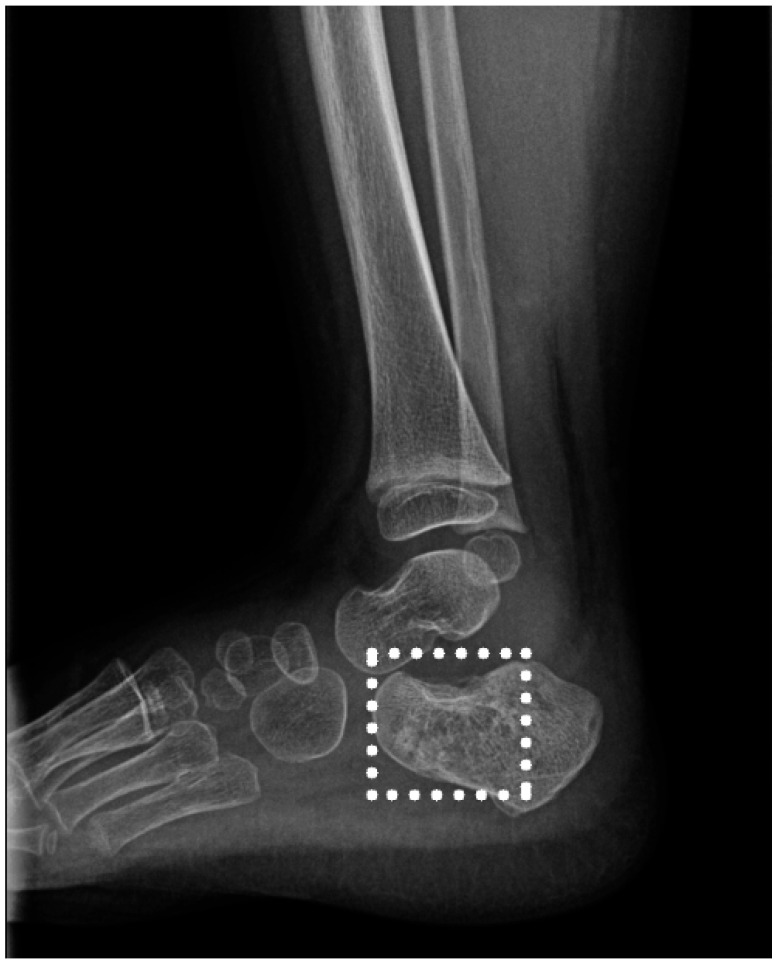
Right ankle X-ray: demineralization and irregular areas of osteolysis and osteocondensation at the level of the calcaneus. The square indicates the lession area.

A 2-year-old female patient, T.I., was brought to the hospital displaying pain and limping associated with her right ankle. The symptoms emerged gradually, with the child avoiding weight on the affected limb and showing discomfort during movement. There was a prior suspicion of synovitis in her right hip. The patient was BCG-vaccinated. Clinical and imaging evaluations led to her urgent admission under a diagnosis of right ankle arthritis (Figure 6).

The treatment involved surgical intervention under general anesthesia, which included incision, lavage, and drainage of the affected ankle, succeeded by stabilization using a below-knee cast. The surgery was successful, and the postoperative period was uncomplicated. Histological analysis of the ankle showed no signs of active inflammation but noted significant capillary hyperemia. The patient was subsequently discharged in a stable and fever-free condition.

Nine days post-operation, the cast was removed, and the ankle was treated with local cleansing and appropriate dressings. She responded well to the procedure, and continued physical rest was advised. Follow-up assessments indicated a favorable recovery trajectory.

However, she was later readmitted with a new diagnosis of right calcaneal osteoarthritis. During this admission, an incisional biopsy was taken for culture, antibiotic sensitivity testing, and histopathological review. Treatment comprised antibiotics and anti-inflammatory drugs. The patient was treated with Cefort (ceftriaxone) and gentamicin, as part of the antibiotic regimen administered during hospitalization, 75 mg/kg/day, administered in divided doses every 12 h, while gentamicin is commonly dosed at 7.5 mg/kg/day, every 12 h doses. These medications were given alongside anti-inflammatory treatment to manage the infection. The treatment aimed to address the osteoarticular complications observed in the calcaneum, with daily wound care contributing to the favorable local evolution. Histopathology revealed active osteomyelitis characterized by giganto-epithelioid granulomatous formations, prompting tests for an infectious etiology, particularly MT. 

Stable clinical improvements were observed in subsequent visits, and a positive Quantiferon test bolstered the suspicion of TB. Despite monthly monitoring showing no significant changes in clinical or radiographic signs, further investigative MRI imaging was advised due to the stagnant nature of her condition.

Despite the lack of microbial growth in cultures and unremarkable histological findings, the patient’s condition remained unimproved. Given the static radiographic findings and normal hematological parameters, a referral to a TB specialist for targeted treatment was suggested, considering the ineffectiveness of the conventional antibiotic regimen.

### 3.4. Case 4

**Figure 7:**
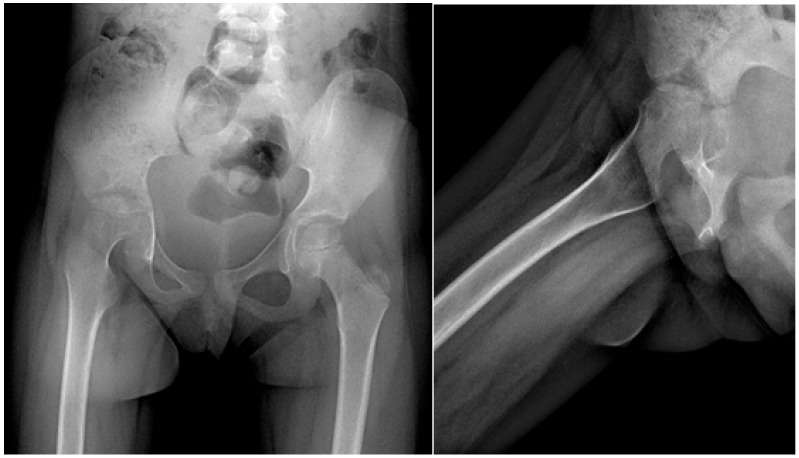
Coxo-femoral arthritis with *Mycobacterium tuberculosis*.

A 6-year-old female, referred to as P.A., presented to the hospital exhibiting a limp (Figure 7). The coinciding pandemic delayed diagnostic procedures. P.A. had a preliminary diagnosis of coxofemoral arthritis potentially caused by MT, supported by clinical and radiological assessments. Notably, she had not been vaccinated with the BCG vaccine.

The patient underwent an incisional biopsy of the right coxofemoral joint, along with joint lavage and drainage under general anesthesia. The surgery proceeded without complications, and subsequent microbiological tests failed to identify any pathogenic organisms or acid-fast bacilli. Histopathological analysis of the joint capsule, periosteum, and fibrous tissue samples revealed chronic granulomatous inflammation characterized by giganto-epithelioid granulomas, Langhans cells, and caseous necrosis. A treatment regimen of ethambutol, isoniazid, pyrazinamide, and rifampicin was initiated in accordance with the national guideline.

P.A. was discharged in a stable condition and returned for routine postoperative care including cast adjustments and wound management. Three months following surgery, she exhibited persistent restrictions in joint mobility, leg length discrepancy, and an antalgic gait. 

Further follow-up appointments revealed a post-surgical scar on the right hip, continued leg length disparity, and restricted hip mobility, which necessitated ongoing physiotherapy and preventive measures against local trauma. The interdisciplinary team continued regular monitoring.

At the two-year mark post-operation, P.A. displayed joint stiffness, minimal rotational movement of the hip, ongoing leg length inequality, and absence of joint pain. The comprehensive management plan included sustained physiotherapy and routine specialist evaluations to address these chronic conditions.

### 3.5. Case 5

**Figure 8:**
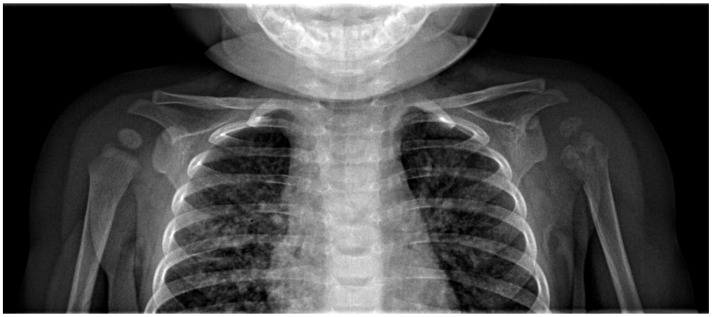
X-ray of the clavicle and left shoulder: demineralization and irregular areas of osteolysis at the diaphyseal-metaphyseal and epiphyseal level, proximal to the left humerus.

A 6-month-old female infant, T.G., presented at the hospital with pain and functional limitations in her left shoulder, characterized by discomfort upon elevating the arm, though without any preceding trauma (Figure 8). The child had undergone BCG vaccination without typical scar development. Preliminary clinical and radiographic evaluations indicated arthritis in the left shoulder, leading to the application of a Dessault bandage for immobilization. Initial recommendations for hospitalization were declined.

**Figure 9:**
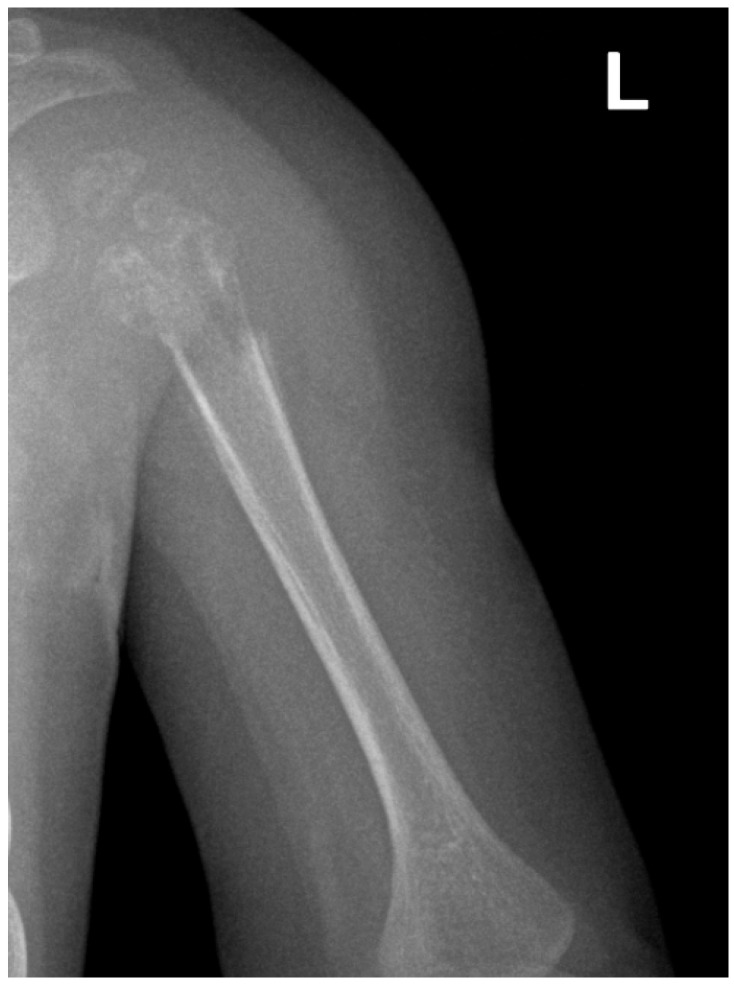
X-ray of the left shoulder: demineralization and irregular areas of osteolysis at the diaphyseal-metaphyseal and epiphyseal level, proximal of the left humerus. L indicates left side of patient.

The infant was admitted two days later following persisting symptoms. She was in a satisfactory overall condition with no external signs of inflammation or injury to the left upper limb, but demonstrated restricted shoulder mobility. An ultrasound did not reveal any pathological findings, yet blood tests indicated anemia and an inflammatory response, prompting the initiation of antibiotic therapy. Although clinical symptoms slightly improved, the inflammatory markers remained elevated. Subsequent imaging prompted surgical exploration on 30 September 2021, targeting the proximal third of the left humerus (Figure 9). The surgery exposed cortical thinning, fragile tissue structures, and minor purulence. Tissue biopsies were conducted for culture and histopathological assessment. Postoperative recovery saw clinical improvement and wound healing, albeit without radiological progress.

**Figure 10:**
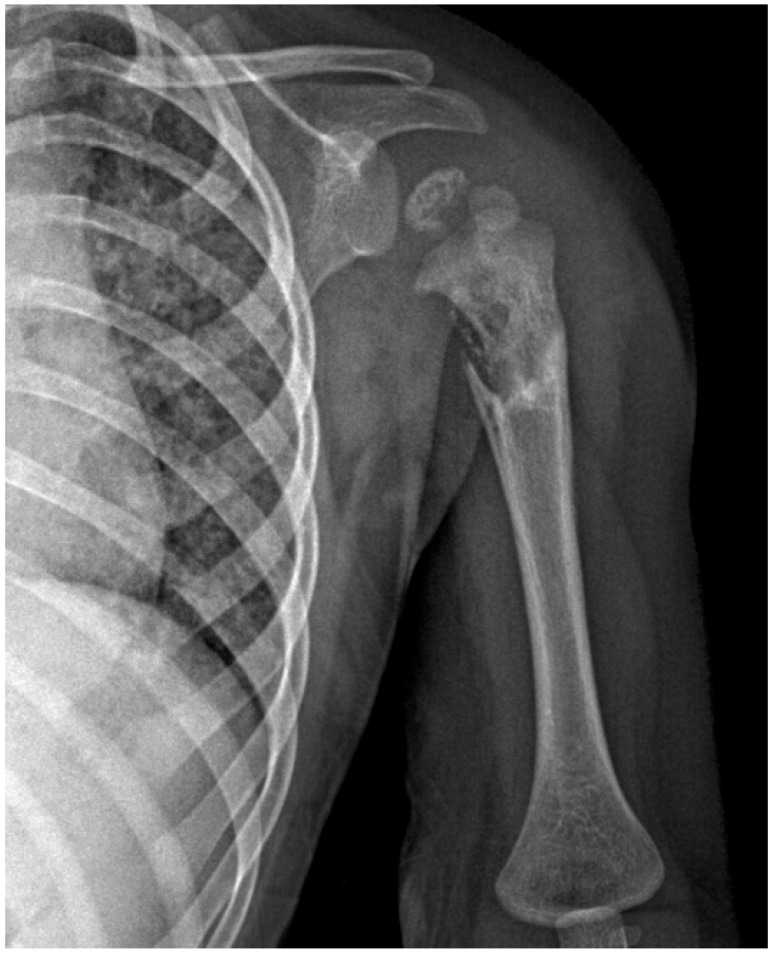
X-ray of the left shoulder: demineralization and irregular areas of osteolysis, with loss of uniform outline at the diaphyseal-metaphyseal and epiphyseal level, proximal of the left humerus.

Two months later, re-admission and further surgical procedures were necessitated by a diagnosis of osteomyelitis in the same region of the humerus (Figure 10). The operations included incision, drainage, lavage, and the placement of antibiotic-impregnated beads. Histology confirmed granulomatous inflammation consistent with TB. Investigations into the patient’s familial contacts revealed the source of infection as the father, who was diagnosed with active pulmonary TB. The child was subsequently managed by the pneumophthisiology department, where TB-specific treatment was commenced.

Despite surgical and pharmacological interventions leading to clinical improvement, the imaging abnormalities persisted. This case underscores the critical need to consider TB in differential diagnoses for unexplained symptoms and treatment-resistant osteoarticular conditions, particularly in young children.

The patient initially received antibiotic treatment with Ampiplus (ampicillin/sulbactam) 900 mg/day intravenously for 8 days, followed by a switch to meropenem for 14 days after surgical intervention. Additionally, gentamicin was used postoperatively, particularly in the form of antibiotic beads placed during the second surgery. The combination of gentamicin, Cefort (ceftriaxone), and meropenem was administered to manage the osteoarticular infection. The patient was later transferred to a specialized pneumology department for further management after the diagnosis of tuberculous osteomyelitis was confirmed. The patient was treated according to the national guidelines.

## 4. Discussion

The findings of this study underscore the complex nature of skeletal tuberculosis (TB) in the pediatric population, particularly its association with ososteomyelitis. The clinical characteristics, vaccination status, and diagnostic findings for the five pediatric skeletal TB cases are summarized in Table S1: Comparative Overview of Pediatric Skeletal TB Cases which can be found in Supplementary Materials. Our investigation into pediatric skeletal TB aligns with the results of several recent studies that highlight the diverse clinical manifestations, diagnostic challenges, and varied treatment approaches associated with this condition. The present study provides valuable insights into how these factors influence disease outcomes, treatment strategies, and long-term prognosis.

### 4.1. Clinical Manifestations and Diagnostic Challenges

Skeletal TB in children presents with a broad range of symptoms, making early diagnosis challenging. Similar to findings reported in the study from Southern Turkey [17], which described variable clinical presentations ranging from joint pain and swelling to neurological deficits in spinal cases, our study also observed a wide range of manifestations, which complicates prompt diagnosis and treatment initiation. The difficulty in achieving an early diagnosis is compounded by the often nonspecific clinical and radiological findings in children, as noted in studies focused on spinal TB [18,20].

Diagnostic tools such as MRI and advanced assays like the Xpert MTB/RIF test have proven valuable in identifying extrapulmonary TB, including skeletal forms [13]. Our study reinforces these findings by showing that integrating these diagnostic modalities can significantly improve early detection rates, particularly in resource-limited settings where conventional microbiological tests may lack sensitivity. This is consistent with prior research that suggests a combination of radiological and molecular diagnostic methods may enhance diagnostic accuracy in pediatric populations.

The cases presented underscore the heterogeneous clinical manifestations of pediatric skeletal TB, a well-documented challenge in the literature. For instance, Case 1, who initially presented with symptoms resembling acute osteomyelitis, reflects the often-ambiguous presentation of TB in children. Studies suggest that pediatric skeletal TB can present with non-specific symptoms, such as pain, swelling, and decreased range of motion, which can easily be confused with more common conditions like pyogenic arthritis or trauma; tuberculous osteomyelitis often presents with non-specific symptoms, such as pain and swelling, which can resemble more common conditions like osteomyelitis or pyogenic arthritis [3]. Similarly, Case 2 and Case 3 illustrate how initial diagnoses of osteomyelitis or synovitis delayed the consideration of TB as a potential cause. A high index of suspicion is critical for early diagnosis in regions with a high TB burden, as noted in studies emphasizing the importance of clinical awareness in endemic areas [20].

The diagnostic overlap between TB and other musculoskeletal conditions is particularly evident in cases like hip TB. The case of hip TB in our study underscores the diagnostic complexities inherent in pediatric skeletal TB, where clinical presentations can mimic other conditions such as developmental disorders. Similar to the multifactorial approach recommended for diagnosing Developmental Dysplasia of the Hip (DDH), where multiple risk factors are assessed for early intervention, pediatric osteoarticular TB requires a comprehensive diagnostic strategy that incorporates clinical, radiological, and microbiological data. This can significantly enhance diagnostic accuracy and ensure timely treatment, as seen in the hip TB cases in our study. Accurate identification of hip involvement through a multidisciplinary approach combining clinical history, imaging, and microbiology can significantly improve patient outcomes by preventing misdiagnosis and ensuring timely treatment [36].

Furthermore, in multifocal TB, where TB affects multiple joints, the delayed recognition of the disease is a significant challenge, a diagnostic issue also noted in the study on multifocal osteoarticular TB in children, which noted that appendicular (hands and feet) involvement was more common than axial (spinal) involvement, while concomitant chest involvement was uncommon [28]. The overlap between TB and more common musculoskeletal infections requires a high degree of suspicion, especially in TB-endemic regions.

### 4.2. Diagnostic Methods

Our use of advanced imaging techniques such as MRI, CT, and X-ray played a pivotal role in diagnosing osteoarticular TB, particularly in Cases 1 and 5. This is consistent with findings from the Southern Turkey study, which highlighted the importance of MRI in detecting bone destruction and abscess formation [17].

The integration of PET/CT, as documented in the 2018 study on the use of 18F-FDG PET/CT for diagnosing osteoarticular TB, could further improve diagnostic accuracy in complex cases like multifocal TB. Advanced imaging modalities such as PET/CT, although not used in our cases, may offer more precise detection of TB activity in challenging presentations [25].

Additionally, the use of molecular diagnostic tests like Xpert MTB/RIF in confirming TB, especially when microbiological cultures are negative like in all of the cases presented, could have provided rapid confirmation and drug-resistance testing if applied, mirrored by the results from the 2016 study on diagnostic accuracy in musculoskeletal infections [13].

Recent advances in molecular diagnostics have greatly enhanced the detection of tuberculosis, particularly in extrapulmonary cases like osteoarticular TB. MacLean et al. (2020) highlight that nucleic acid amplification tests (NAATs), such as the Xpert MTB/RIF assay, are increasingly used to not only detect TB but also identify drug-resistant strains [14]. These molecular tests provide rapid and accurate results, which are critical for initiating timely treatment and preventing disease progression. The use of the Xpert MTB/RIF test aligns with these findings, as it played a crucial role in diagnosing TB in cases where traditional methods like culture were inconclusive. Xpert MTB/RIF Ultra has shown improved diagnostic accuracy, even with less invasive sample collection methods like oral swabs [37].

Urine-based LAM antigen tests have emerged as a valuable tool for diagnosing TB in pediatric patients, especially in low-resource settings [38].

The C-Tb skin test has been evaluated as a safer and more effective alternative to traditional tuberculin skin tests, particularly in pediatric TB cases [39].

### 4.3. Treatment Approaches and Outcomes

In managing osteoarticular tuberculosis, various treatment modalities were employed across the cases presented. The mainstay of treatment involved a combination of surgical intervention and anti-tuberculosis therapy tailored to each patient’s condition, accompanied by regular follow-up for monitoring treatment efficacy and disease progression.

Pediatric TB management according to the WHO emphasizes early diagnosis through clinical evaluation, history of TB contact, and imaging techniques like chest X-rays. Diagnostic tests, such as GeneXpert, and non-invasive specimen collection, like stool or gastric aspirates, are recommended. For drug-susceptible TB, the WHO suggests a treatment regimen of 2 months with HRZE followed by 4 months of HR, extending to 12 months for severe cases like osteoarticular or CNS TB. Multidrug-resistant TB prevention includes levofloxacin-based treatments. TB preventive therapy and BCG vaccination are crucial, especially in high-risk children, with ongoing follow-up to monitor treatment response and prevent relapse [40].

The Romanian TB guidelines emphasize a multidisciplinary approach to managing extrapulmonary TB cases, including osteoarticular TB, which is one of the more common forms of TB outside the lungs. Diagnosing osteoarticular TB in pediatric patients requires a thorough evaluation, combining clinical, radiological, bacteriological, and histopathological data. The diagnostic process is challenging due to the disease’s slow progression and non-specific symptoms in children, necessitating the exclusion of other potential pathologies. For pediatric cases, imaging tools like X-rays and MRI, alongside bacteriological confirmation (such as smear tests for acid-fast bacilli or culture) and histopathological examination, are recommended for an accurate diagnosis [41].

The treatment of pediatric osteoarticular TB follows the standard anti-TB regimen, typically consisting of isoniazid (H), rifampicin (R), pyrazinamide (Z), and ethambutol (E) in an intensive phase of two months, followed by a continuation phase of isoniazid and rifampicin for at least four more months. However, in cases involving skeletal TB, including osteoarticular TB, the treatment duration may be extended up to 12 months, depending on the severity and progression of the disease. For osteoarticular TB, surgical intervention may be necessary in cases of severe joint involvement or when there is a risk of permanent deformity. The guidelines stress the importance of monitoring drug side effects, especially in children, where the potential for ethambutol-induced optic neuritis requires careful ophthalmologic evaluation during treatment. The guidelines also emphasize long-term follow-up, particularly in pediatric cases, to detect complications such as joint deformities, growth disturbances, or recurrence of TB. Given the slower progression and complex nature of osteoarticular TB, the follow-up period is recommended to be at least two years to ensure full recovery and address any long-term issues related to skeletal development [41].

Surgical management played a pivotal role, with procedures like surgical debridement and drainage being commonly used, particularly in cases where significant joint or bone involvement was observed. For instance, in Case 1, fistula formation required debridement and excision, while Case 5 underwent multiple surgical procedures for osteomyelitis. Incision and drainage were performed in several cases, such as Case 2 and Case 3, to manage abscesses and infected tissue. Additionally, joint lavage and biopsy were essential in cases with severe joint involvement, like Case 3 and Case 4, as these procedures allowed for diagnostic confirmation of tuberculosis and relief of inflammatory symptoms. Immobilization was also crucial in all cases involving weight-bearing joints, where post-surgical measures, such as casts or bandages, were applied to reduce pressure and allow for proper healing.

Pharmacological therapy, particularly multidrug antituberculous treatment, was a cornerstone of the treatment approach. All patients received a regimen consisting of isoniazid, rifampicin, pyrazinamide, and ethambutol, which is standard in the management of tuberculosis. In Case 1, this regimen proved critical for treating osteoarticular TB and was adjusted as necessary based on the disease course. Additionally, antibiotics like ceftriaxone were used initially in some cases to control suspected bacterial infections, as seen in Case 1 and Case 5, before TB was confirmed and the patient started targeted therapy.

Outcomes were generally positive, with most patients showing improvement in response to the multidrug therapy combined with surgical interventions. In Case 1, after adjusting to the four-drug regimen, the patient showed gradual improvement, despite complications such as fistula formation. Similarly, Case 5 demonstrated significant clinical improvement after surgery and targeted therapy, although persistent imaging abnormalities remained. However, complications were present in some cases, such as long-term sequelae like leg length discrepancies and joint stiffness observed in Case 4. These complications required continuous monitoring, physiotherapy, and preventive strategies to maintain joint function and manage growth issues.

Findings from the 2019 study on spinal TB emphasized the effectiveness of posterior-only approaches using titanium mesh cages for stabilizing the spine in pediatric patients [22]. In juvenile spinal tuberculosis, non-surgical treatment is often the preferred first-line approach, especially in children under the age of 10. However, surgical intervention may be necessary in cases with progressive neurological deficits or worsening kyphosis. Surgical treatment can achieve satisfactory results in selected cases, though common complications, such as postoperative proximal or aggravated kyphosis deformities, are more frequent in younger patients with multiple lesion segments. These outcomes underscore the importance of careful patient selection and surgical planning to minimize complications while ensuring effective treatment. This is particularly relevant to cases like Case 5, where the young age of the patient and the extent of the lesion might influence the choice of surgical intervention. [26]. The study on one-stage surgical management of spinal TB by anterior decompression and posterior instrumentation suggests a potentially more effective approach. This single-stage method, which combines anterior decompression with posterior stabilization, could provide effective and feasible relief of spinal cord compression and reduced the need for additional surgeries [31]. One-stage management, as highlighted in the study, offers multiple advantages, including shorter hospital stays, reduced surgical stress, and fewer postoperative complications. The application of this method in spinal TB cases might allow for better long-term outcomes by addressing both decompression and stabilization in a single intervention, minimizing the risk of spinal deformities and neurological deficits.

Furthermore, the one-stage approach is particularly beneficial in resource-constrained settings, where access to multiple surgeries may be limited. The reduced need for prolonged follow-up also aligns with our findings, where consistent long-term follow-up was a challenge in ensuring the complete recovery of TB patients.

### 4.4. Treatment Complexity and Multidisciplinary Management

The cases illustrate the complexity of treating pediatric skeletal TB, requiring a multidisciplinary approach involving orthopedic surgeons, pediatricians, infectious disease specialists, and physiotherapists. Treating osteoarticular tuberculosis is complex, requiring precise surgical interventions, accurate diagnostics, and prolonged multidrug therapy. Cases like Case 1 and Case 3 highlight the diagnostic challenges due to overlapping symptoms with osteomyelitis, necessitating repeated surgeries and thorough histopathological evaluation.

Pharmacological management, involving a long-term anti-tuberculosis regimen, required close monitoring for efficacy and side effects, as seen in Case 2 (R.M.) and Case 4 (P.A.). Adherence to therapy was essential for preventing drug resistance and ensuring recovery.

Multidisciplinary collaboration was key to success, involving orthopedic surgeons, infectious disease specialists, radiologists, and physiotherapists. For example, in Case 3, advanced imaging and referrals were necessary for ongoing treatment decisions, and physiotherapists played a crucial role in long-term rehabilitation. Overall, this coordinated approach was vital for managing the complexity of osteoarticular TB. Similarly, in Case 5 (6-month-old female), multiple surgical debridements were required alongside medical therapy, underscoring the need for individualized treatment plans and regular multidisciplinary evaluations.

Comparatively, findings in the literature emphasize that surgical intervention, combined with antituberculous chemotherapy, offers the best outcomes in cases of advanced disease or when conservative treatment does not yield satisfactory results. The cases align with this approach, demonstrating that combining drug therapy with surgery helps mitigate long-term functional impairments and prevents recurrence.

In our study, no serious adverse reactions (SAR) were observed among the patients during the course of treatment. Despite the use of multidrug anti-TB therapy, including isoniazid, rifampicin, pyrazinamide, and ethambutol, none of the patients experienced significant hepatotoxicity, nephrotoxicity, or other notable complications commonly associated with these medications.

### 4.5. Long-Term Outcomes and Follow-Up

Long-term follow-up is crucial in managing pediatric osteoarticular TB, as seen in our cases, particularly for monitoring potential relapses and growth disturbances. In Case 5, involving spinal TB, consistent follow-up was necessary to detect and address residual spinal deformities. The South African cohort study on spinal TB similarly found that inadequate follow-up was associated with poor long-term outcomes, particularly in children who experienced relapse or complications [18].

In addition, the 20-year retrospective study from a third-level referral center emphasized the importance of follow-up in preventing long-term complications such as joint stiffness and limb length discrepancies, which were also observed in our cases [20].

Our findings also emphasize the importance of long-term follow-up, particularly to monitor for complications like joint deformities and spinal curvature. Developing standardized protocols for post-treatment care, especially in resource-limited regions, is critical for preventing recurrence and ensuring the complete rehabilitation of pediatric TB patients.

### 4.6. Socioeconomic Factors and Health System Limitations

The cases also highlight the impact of socioeconomic factors and health system limitations on the management of pediatric TB. Case 4 and Case 5 exemplify how delays in diagnosis and treatment can occur due to external factors like the COVID-19 pandemic, which has been shown to disrupt healthcare access and exacerbate TB management challenges. This reflects findings in the South African cohort, where access to healthcare was a significant challenge in managing long-term follow-up for pediatric TB cases [18]. Addressing these systemic barriers is essential to improving outcomes for pediatric TB patients.

Adherence to long-term, complex TB treatment regimens is challenging, especially in pediatric populations, as evidenced by Case 1, where the patient initially left against medical advice. Studies suggest that such adherence challenges are more prevalent in lower-income settings or situations where follow-up care is difficult to ensure or due to limited healthcare access. This lack of consistent care can lead to complications and relapses, as ongoing monitoring is essential for managing TB. Additionally, health system limitations, such as inadequate resources, delays in specialized referrals, or insufficient screening programs for TB, further complicate management.

As healthcare systems evolve, telemedicine offers promising potential for managing pediatric skeletal tuberculosis remotely. By enabling continuous monitoring and reducing the need for frequent hospital visits, telemedicine can help address the challenges posed by long-term follow-up in resource-limited areas. Telemedicine has already demonstrated success in managing other chronic conditions, suggesting it could play a crucial role in tuberculosis care moving forward [42,43].

### 4.7. Overlapping Features of TB and Osteomyelitis

The cases further emphasize the diagnostic overlap between TB and bacterial osteomyelitis, a theme frequently discussed in the literature. Case 2 and Case 3 illustrate how skeletal TB can initially present similarly to bacterial osteomyelitis, delaying appropriate TB-specific treatment. Any case of osteomyelitis that fails to respond to standard antibiotics should raise suspicion for TB, especially in endemic areas or where atypical radiological features are present. The literature supports the need for including TB in the differential diagnosis of osteoarticular infections, particularly in young children, where classic TB symptoms may be absent [29].

Osteomyelitis can mimic or obscure the diagnosis of skeletal TB, as seen in some cases where initial misdiagnosis delayed appropriate TB treatment. Our findings align with studies like those by Rasool (2001), which described the overlapping clinical features of pyogenic and fungal infections, bone tumors, and TB in pediatric populations, highlighting how the nonspecific nature of early symptoms often leads to delays in TB diagnosis [8]. Similarly, the study by Shikhare et al. (2011) emphasized the difficulty of distinguishing TB from other infections like osteomyelitis, particularly in the early stages when radiological findings are inconclusive [7]. Differentiating between TB and other infections and malignancies/metastases remains essential for directing appropriate therapy, especially in pediatric patients, where the consequences of delayed treatment can be severe.

Furthermore, the study on arthroscopically assisted treatment of knee joint TB provides additional insights into how advanced diagnostic techniques, such as arthroscopy, can be used to differentiate between TB and other musculoskeletal infections [30]. Incorporating such diagnostic tools could potentially reduce diagnostic delays in challenging cases like those presented in our study.

The study also highlights that the association between TB and osteomyelitis may be more common in settings with high TB endemicity or in immunocompromised children. This finding necessitates a high index of suspicion for TB in all pediatric patients presenting with osteomyelitis, especially when initial treatments do not lead to improvement.

### 4.8. Limitations and Future Directions

This study is limited by its retrospective design and the relatively small sample size, which may not capture the full spectrum of skeletal TB manifestations in children. Future research should focus on prospective studies with larger cohorts to validate these findings and explore the potential benefits of new diagnostic and therapeutic approaches, including the role of novel imaging techniques and immunological assays in early diagnosis. Additionally, more research is needed to understand the long-term impacts of pediatric skeletal TB on growth, development, and quality of life, particularly in relation to different treatment modalities.

Future research should focus on refining diagnostic tools like molecular assays and imaging techniques to improve sensitivity and specificity, especially in low-resource settings. Long-term studies evaluating the outcomes of different surgical techniques, such as one-stage versus multi-stage interventions, would also provide further insights into optimizing treatment for pediatric spinal TB.

This study’s strength lies in integrating findings from multiple studies; our research contributes to a growing understanding of how best to tackle this challenging condition, particularly in resource-constrained settings where TB remains a significant public health concern.

In our study, BCG vaccination status was documented in four of the five cases, and no definitive pattern emerged in terms of its protective effect against osteoarticular TB. These limited data highlight the need for further investigation into the specific role BCG plays in preventing skeletal forms of TB. While BCG vaccination is known to reduce the risk of severe TB manifestations, including miliary TB and TB meningitis, its impact on osteoarticular TB remains less clear. Future research with larger datasets could provide more clarity on this issue.

## 5. Conclusions

This study highlights the critical need for early and accurate diagnosis, individualized treatment approaches, and comprehensive follow-up in managing pediatric skeletal TB, emphasizing the utility of advanced imaging and molecular diagnostics to reduce diagnostic delays. Our findings also underline the importance of integrating multidisciplinary treatment strategies to improve long-term outcomes. The study underscores the necessity for a multidisciplinary approach involving orthopedic surgeons, infectious disease specialists, and radiologists to ensure optimal patient management.

Given the increasing burden of TB globally, particularly in children, our study underscores the necessity for enhanced public health policies targeting early screening, risk-based interventions, and improved access to healthcare for vulnerable populations. Future research should focus on improving early detection methods, such as the integration of more sensitive molecular diagnostics and imaging technologies. Additionally, prospective studies examining the long-term impacts of different surgical interventions and their timing relative to anti-TB therapy could provide valuable insights into optimizing treatment protocols. Addressing the socioeconomic factors that hinder timely diagnosis and treatment access is also a key area for further investigation. These findings highlight the need for continued refinement of diagnostic and therapeutic approaches to better manage pediatric TB and reduce the burden of long-term complications.

## Figures and Tables

**Table 1:** Characteristics of the selected articles.

Article Title	Publication Year	Area of Interest	Number of Patients and Ages	Purpose
Skeletal Tuberculosis in Pediatric Population for 15 Years; Twenty Cases from Southern Turkey [17]	2023	Skeletal Tuberculosis	20 patients; 7 months–16 years old	To evaluate the clinical characteristics, diagnostic methods, treatment, and prognosis of skeletal tuberculosis
Loss to long-term follow-up in children with spinal Tuberculosis: a retrospective cohort study at a tertiary hospital in the Western Cape, South Africa [18]	2022	Spinal Tuberculosis	32 patients; 1–14 years old	To investigate compliance with long-term follow-up in pediatric patients with spinal tuberculosis
Treatment of Stage I-III Hip Joint Tuberculosis with Open Surgical Debridement and Hip Spica in Children: A Retrospective Study [19]	2022	Extraspinal Skeletal Tuberculosis	87 patients; 2–14 years old	To evaluate the outcome of the combination of surgical debridement and hip spica immobilization in the treatment of hip joint tuberculosis
A 20-year retrospective study of osteoarticular Tuberculosis in a pediatric third level referral center [20]	2021	Spinal Tuberculosis	159 patients; 8 months–16 years old	To describe the clinical manifestations, radiological aspects, diagnostic modalities, and therapeutic approach to osteoarticular tuberculosis
The outcome of intervertebral surgery in the treatment of lumbar Tuberculosis in children: A case series and long-term follow-up [21]	2019	Spinal Tuberculosis	18 patients; 5–10 years old	To review the clinical manifestations and investigate the clinical feasibility and efficacy of intervertebral surgery in the treatment of lumbar tuberculosis
Comparison of mid-term outcomes of posterior or postero-anterior approach using different bone grafting in children with lumbar Tuberculosis [22]	2019	Spinal Tuberculosis	51 patients; 2–17 years old	To compare the results of the posterior surgical approach with the combined (anterior and posterior) approach using different bone grafts in the treatment of lumbar tuberculosis
The efficiency of the posterior-only approach using shaped titanium mesh cage for the surgical treatment of spine Tuberculosis in children: A preliminary study [23]	2018	Spinal Tuberculosis	22 patients; 4–16 years old	To evaluate the efficiency of the exclusively posterior surgical approach using a titanium mesh cage in the treatment of spinal tuberculosis
Mycobacterium bovis Osteitis Following Immunization with Bacille Calmette-Guérin (BCG) in Korea [24]	2018	Extraspinal Tuberculosis BCG Osteitis	21 patients; 6 months–3 years old	To analyze the clinical characteristics of tuberculosis caused by BCG immunization
Osteoarticular Tuberculosis in children. A fast reappearing disease diagnosed by 18F-FDG PET/CT and other modalities. The cover page of Nicholas Andry booklet L’Orthopedie [25]	2018	Skeletal Tuberculosis	Review	To review the imaging correlates discovered and provide imaging for the diagnosis of osteoarticular tuberculosis
Diagnostic Accuracy of the Xpert MTB/RIF Assay for Extrapulmonary Tuberculosis in Children with Musculoskeletal Infections [13]	2016	Skeletal Tuberculosis	102 patients; 2–9 years old	To investigate the accuracy of the Xpert MTB/RIF test for the diagnosis of extrapulmonary tuberculosis
Treatment effect, postoperative complications, and their reasons in juvenile thoracic and lumbar spinal Tuberculosis surgery [26]	2015	Spinal Tuberculosis	54 patients; 2–18 years old	To evaluate treatment effects, complications and reasons for focal debridement, correction of deformities, fusion of bone grafts, and internal fixation in spinal tuberculosis
One-year multidrug treatment for Tuberculosis of the cervical spine in children [27]	2015	Spinal Tuberculosis	21 patients; 2–12 years old	To review the clinical and radiological features and treatment outcomes in spinal tuberculosis
Multifocal osteoarticular Tuberculosis in children [28]	2011	Multifocal osteoarticular Tuberculosis	16 patients; 1–14 years old	To analyze the clinical manifestations and therapeutic evolution of multifocal osteoarticular tuberculosis
Tuberculosis of the foot and ankle in children [29]	2011	Extraspinal Skeletal Tuberculosis	21 patients; 3–14 years old	To describe the clinical manifestations and management of foot and ankle tuberculosis
Arthroscopically assisted treatment of adolescent knee joint Tuberculosis [30]	2010	Extraspinal Skeletal Tuberculosis	41 patients; 7–16 years old	To evaluate the role of the knee arthroscopy in the diagnosis and treatment of tuberculosis of the knee
One-stage surgical management for children with spinal Tuberculosis by anterior decompression and posterior instrumentation [31]	2009	Spinal Tuberculosis	15 patients; 5–16 years old	To analyze the effectiveness of the surgical approach in a single intervention (anterior decompression, bone grafting, posterior vertebral arthrodesis, and segmental instrumentation), to evaluate the results of this method, and to verify the importance of early reconstruction for spinal stabilization in spinal tuberculosis

## Data Availability

The original contributions presented in the study are included in the article and Supplementary Materials, further inquiries can be directed to the corresponding author.

## Supplementary Materials

The following supporting information can be downloaded at: https://www.mdpi.com/article/10.3390/children11111279/s1, Table S1: Comparative Overview of Pediatric Skeletal TB Cases.
