# Supplementary material for: Reevaluating Pediatric Osteomyelitis with Osteoarticular Tuberculosis: Addressing Diagnostic Delays and Improving Treatment Outcomes
Source: Children (Basel). 2024 Oct 23;11(11):1279. doi: 10.3390/children11111279 (PMC11592565; doi:10.3390/children11111279)
Supplement: Supplementary file 1 [file children-11-01279-s001.zip › children-3241285-supplementary.pdf]

Table S1. Comparative Overview of Pediatric Skeletal TB Cases.

	Case I	Case II	Case III	Case IV	Case V
Gender	masculine	masculine	feminine	feminine	feminine
Age at first presentation	12 years and 3 months	1 years and 11 months	2 years and 1 month	6 years and 5 months	6 months
Age at diagnosis of TB	12 years and 3 months	2 years and 7 months	2 years and 4 months	6 years and 5 months	9 months
MT infection localization.	Left hip	Left knee	calcaneus	Right hip	Left shoulder
Fever	no	no	no	no	no
Pain	yes	yes	yes	yes	yes
Affected bone growth	no	no	no	yes	no
BCG vaccine	yes	yes	yes	no	yes
Score/Scar after vaccination	Score+ scar -	Score+ scar -	Score+ scar -	-	Sore+ scar -
IDR PPD (at 72 hours)	15 mm	unavailable	unavailable	8 mm	Did not undergo
QuantiFERON test	positive	positive	positive	positive	positive
Anemia	yes	yes	yes	no	yes
Inflammation markers	VSH ↑ PCR ↑	VSH ↑ PCR N	VSH N PCR N	VSH ↑ PCR ↑	VSH ↑ PCR ↑
HIV infection	no	no	no	no	no
Histopathology	Suggestive for MT.	Suggestive for MT.	Suggestive for MT.	Suggestive for MT.	Suggestive for MT.
Microbiology with Ziehl-Neelsen	BAAR negative	BAAR negative	BAAR negative	BAAR negative	BAAR negative

Direct contact with identified MT person	unidentified	yes	unidentified	unidentified	yes
